# Bioavailable Sulforaphane Quantitation in Plasma by LC–MS/MS Is Enhanced by Blocking Thiols

**DOI:** 10.1021/acs.jafc.3c01367

**Published:** 2023-08-16

**Authors:** Rachel S. Grady, Tinna Traustadóttir, Anthony F. Lagalante, Aimee L. Eggler

**Affiliations:** † Department of Chemistry, Villanova University, Villanova, Pennsylvania 19085, United States; ‡ Department of Biological Sciences, Northern Arizona University, Flagstaff, Arizona 86001-5766, United States

**Keywords:** sulforaphane, broccoli sprouts, plasma concentration, reversible thiol conjugates, blocking thiols

## Abstract

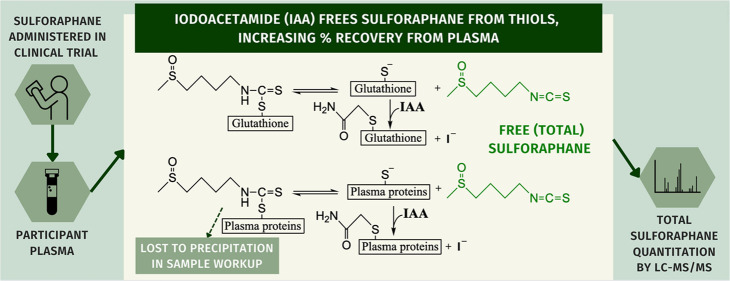

Quantifying sulforaphane (SFN) and its thiol metabolites in biological samples using liquid chromatography–tandem mass spectrometry is complicated by SFN’s electrophilic nature and the facile dissociation of SFN-thiol conjugates. SFN can be lost during sample preparation due to conjugation with protein thiols, which are precipitated and discarded. We observe that only 32 ± 3% of SFN is recovered 2 h after spiking into fetal bovine serum. The SFN-glutathione conjugate prepared at 10 mM in 0.1% formic acid in water (pH 3) dissociated by approximately 95% to free SFN, highlighting the difficulty in preparing thiol metabolite standards. We used the alkylating agent iodoacetamide (IAA) to both release SFN from protein thiols and force the dissociation of SFN metabolites. This thiol-blocking method increased SFN percent recovery from serum from 32 to 94 ± 5%, with a 4.7 nM method limit of quantitation. Applying the method to clinical samples, SFN concentrations were on average 6 times greater than when IAA was omitted. The IAA thiol-blocking method streamlines the analysis of bioavailable SFN in plasma samples.

## Introduction

Sulforaphane (SFN) is a phytochemical derived from cruciferous vegetables, and broccoli sprouts are particularly rich in its precursor, glucoraphanin. First recognized in 1992 as a potent activator of the expression of cytoprotective genes associated with redox balance, detoxification, and cellular defense processes,^1^ SFN quickly progressed through animal models to clinical trials. To date, ClinicalTrials.gov lists 92 clinical trials with SFN, mostly in the form of broccoli sprout preparations, treating a variety of chronic diseases such as Alzheimer’s, cancer, and diabetes. Methods to accurately detect SFN in blood plasma are an essential part of evaluating its clinical use and efficacy. Liquid chromatography–tandem mass spectrometry (LC–MS/MS) is the most sensitive and selective technique to quantify SFN and its metabolites in human plasma. However, analysis is complicated by the reactivity of SFN and the stability of its thiol-conjugated metabolites.

**Figure 1:**
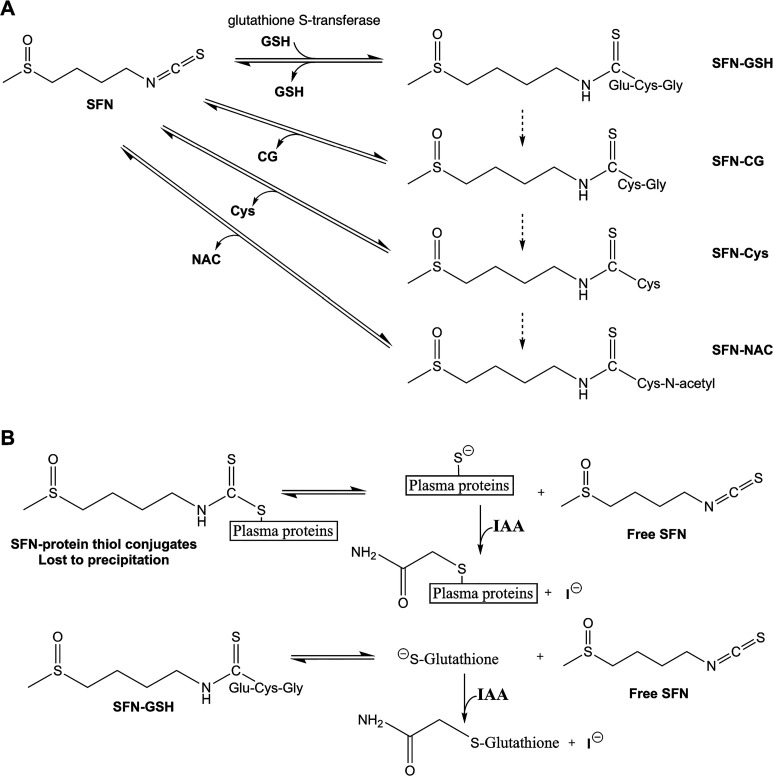
(A) Metabolism of SFN via the mercapturic acid pathway, initiated by addition of glutathione to the isothiocyanate group of SFN. Each reaction is readily reversible to yield free SFN. (B) Method proposed in this work. SFN conjugated to protein thiols is lost upon protein precipitation during sample preparation. Addition of IAA blocks thiols upon the facile dissociation of SFN-thiol conjugates, both protein thiols in plasma and SFN metabolites (SFN-GSH shown as an example). Blocked thiols cannot reconjugate with SFN, freeing SFN for detection.

SFN has an electrophilic isothiocyanate group and thus reacts with a variety of nucleophiles, thiols in particular. After the absorption of SFN into the intestinal lining, it readily reacts with glutathione (GSH), catalyzed by intracellular glutathione S-transferase (Figure 1A). Further enzymatic modifications in the mercapturic acid pathway generate cysteine, cysteine–glycine, and finally N-acetyl cysteine (NAC) conjugates, which are excreted in urine.^3^ The electrophilic isothiocyanate group is also a major means by which SFN acts on its targets, including the C151 residue of Keap1, a primary repressor of the Nrf2 transcription factor.^2,4,5^ Upon escape from Keap1 repression, Nrf2 upregulates cytoprotective gene expression.

A key feature of SFN biological activity is the facile reversibility of its thiol conjugates. Isothiocyanate-thiol metabolites readily dissociate (Figure 1A), even upon simple dilution.^6^ SFN is thus readily freed from a thiol conjugate to act on biological targets. For example, both SFN and its glutathione conjugate induced expression of Nrf2-regulated genes in liver and colon cells.^7^ Indeed, the four thiol conjugates of SFN are biologically active in vivo, likely due to their facile reversion to free SFN, reducing the incidence of carcinogenesis in various mouse models.^8,9^ The reversibility of the SFN-GSH conjugate is also responsible for the ability of SFN to deplete cellular GSH. Free SFN is transported into a cell and conjugated with GSH, and the conjugate is excreted and then dissociates, freeing SFN.^10^ This cycle is repeated, leading to a net reduction in total cellular GSH. Recently, SFN-NAC was shown to readily convert to SFN in rats when administered orally and in plasma, and both had similar antipulmonary fibrotic effects.^11^ In summary, SFN metabolites are readily reversible and yield free, bioavailable SFN.

This rapid dissociation of isothiocyanate-thiol metabolites into free SFN and free thiol under aqueous conditions upon dilution creates potential problems for accurate quantitation of SFN-thiol metabolites by LC–MS/MS. For example, an external calibration curve is typically generated by serially diluting an SFN standard into the aqueous buffer. The significant dissociation of a metabolite means that the concentrations assigned to each peak would be much higher than the actual concentrations of the metabolite in the solution. For example, if a metabolite dissociates to free SFN by 90% during external calibration curve generation, a determined concentration of the metabolite in plasma will be overestimated by a factor of 10. In addition, plasma samples are typically diluted prior to analysis, and if this causes the dissociation of isothiocyanate-thiol metabolites, the actual concentrations of the metabolites in plasma would be underestimated.

An additional factor in the LC–MS/MS quantitation of SFN in plasma is the tendency of SFN to conjugate reactive thiols in plasma proteins (Figure 1B), which could affect the percent recovery when proteins are precipitated during sample preparation. While some reported percent recoveries of SFN spiked in plasma range from 83.3 to 94%,^12−14^ suggesting little loss of SFN, a significantly lower percent recovery of spiked SFN in plasma of only 14–19% was recently reported.^15^ This discrepancy may be due to the amount of time that SFN is incubated in blank plasma or other blank matrices prior to protein precipitation. A meaningful amount of SFN in patient samples may go undetected due to protein thiol conjugation.

A method to rescue SFN conjugated to protein thiols from loss upon protein precipitation could greatly increase the percent recovery of SFN in human plasma. Previous work showed that the SFN-Keap1 C151 conjugate is also highly reversible, and special care was required during mass spectrometry analysis to maintain the conjugate, by reducing sample preparation time and omitting iodoacetamide (IAA) from the protocol.^2^ IAA readily reacts with thiols as the thiol-acetamide product is stable and free iodide is released, rendering the modification irreversible, a process known as thiol blocking. Thus, incubation of SFN-containing plasma samples with IAA for a sufficient time period could be employed to block protein thiols, freeing SFN from plasma proteins and increasing percent recovery (Figure 1B).

Therefore, the first objective of this work was to determine the extent to which the SFN-GSH thiol-conjugate (as a representative SFN metabolite) dissociates upon dilution for LC–MS/MS method development such as external calibration curve generation. Next, the amount of SFN spiked into fetal bovine serum (FBS) that was lost due to protein precipitation during sample workup was determined, using time points over 2 h after adding SFN to serum. The third and main objective of this work was to develop a protocol to quantitate the amount of bioavailable SFN in serum or plasma, using IAA to release SFN from both thiol-conjugate metabolites and plasma proteins (Figure 1B). Finally, this method was applied to quantitate SFN in human plasma obtained from an acute supplementation clinical trial.

## Materials and Methods

### Materials

LC–MS grade acetonitrile and methanol (Optima) were purchased from Fisher Chemical (Thermo Fisher Scientific, Waltham, MA, USA). LC–MS grade formic acid (UltraPure) was from CovaChem (Loves Park, IL, USA). Ethanol (200 proof) was from Pharmco (Brookfield, CT, USA). Reverse osmosis purified Milli-Q water used in LC–MS analysis was from a Millipore water purification system (Merck, Darmstadt, Germany). Analytical standards at >95% purity of SFN, SFN-*d*_8_, SFN-GSH, SFN-Cys, and SFN-NAC were purchased from Toronto Research (Toronto, ON, Canada). IAA (Bioultra, ≥ 99% pure) and l-cysteine (97% pure) were purchased from Sigma-Aldrich (St. Louis, MI, USA). FBS was purchased from Atlanta Biologicals (Flowery Branch, GA, USA). 5,5′-dithio-bis (2-nitrobenzoic acid) (DTNB, Ellman’s Reagent) was at 99.9% purity and purchased from Chem-Impex International (Wood Dale, IL, USA).

### LC–MS/MS MRM Method

SFN, SFN-GSH, SFN-NAC, and SFN-Cys standards were prepared in 0.1% formic acid in water. Compound-specific MS parameters, declustering potential and collision energy, were optimized by using direct infusion of each compound. A SCIEX QTRAP 4500 coupled to a Prominence high-performance liquid chromatography (HPLC) system consisting of binary LC-20AD pumps and a SIL-20A autosampler (Shimadzu, Colombia, MD, USA) was operated in multiple reaction monitoring (MRM) mode with a SCIEX Turbo V source with an electrospray ionization (ESI) probe in positive polarity. Electrospray source parameters were set as follows: EP = 10, CXP = 10, CUR = 30 psi, CAD = medium, ISV = 5500 V, TEM 400 °C, and GS1 and GS2 = 50 psi. LC–MS/MS data were processed using Analyst version 1.7.2. Chromatographic separation was performed on a Phenomenex Kinetex C18 column (2.6 μm, 100 Å, 100 × 4.60 mm^2^) thermostated at 40 °C. Mobile phases consisted of 0.1% formic acid in water (A) and 0.1% formic acid in acetonitrile (B) at a total flow of 0.4 mL/min. Injection volumes were 2 μL. The gradient was programmed as follows: 5% B (1 min hold), ramp to 95% B (1–5 min), and hold at 95% B (5–8 min). For SFN recovery with IAA and human plasma sample analysis, this same method was used with a diverter valve that pumped column eluent to the mass spec from 3 to 8 min to avoid source contamination with excess IAA. The MRM transitions monitored are shown in Table S1.

Formic acid (0.1%) in acetonitrile was chosen as a precipitation solvent because SFN shows high stability in acetonitrile,^16^ in particular compared to protic organic solvents methanol and ethanol, which accelerate the decomposition of SFN due to solvolysis. While trifluoroacetic acid (TFA) is a common method of protein precipitation for the detection of SFN in plasma by mass spectrometry,^12,17,18^ it can cause ion suppression in MS analyses. Solid-phase extraction (SPE) is often employed to remove TFA prior to analysis; however, SFN is unstable in common SPE solvents and under elevated temperatures used during SPE cleanup.

### SFN Dissociation Testing

To test the extent of metabolite dissociation in 0.1% formic acid in water, SFN-GSH (10 mM) was serially diluted to 100 nM with 0.1% formic acid in water (pH 3), with samples kept on ice during preparation. Free SFN was detected by immediately precipitating the remaining SFN-GSH metabolite and free glutathione with acetonitrile, with free SFN remaining in the solution. Free SFN was quantitated by LC–MS/MS MRM using an external standard calibration curve and expressed as a percent of the initial SFN-GSH concentration.

### Assessing the Degree of SFN Conjugation to Thiols in Serum

SFN (534 nM final concentration) was incubated in FBS at room temperature to allow SFN to react with protein thiols. At various time points up to 2 h, 50 μL aliquots were taken, and proteins were precipitated with 200 μL of ice-cold 0.1% formic acid in acetonitrile. Samples were centrifuged for 5 min at 5200*g*, and the supernatant was transferred to an autosampler vial and analyzed with the LC–MS/MS MRM Method as described above. Percent recovery was calculated by comparing the SFN peak area of extracted FBS samples to an equivalent concentration of SFN standard in 0.1% formic acid in acetonitrile corresponding to 107 nM SFN, accounting for dilution by the precipitation solvent. Standard deviation was determined based on triplicate injections of a given sample.

### Free Thiol Quantitation in Serum and Plasma

Quantitation of free thiols (R-SH, sulfhydryl groups) was performed as previously described with minor modifications,^19^ using DTNB. DTNB reacts with free sulfhydryl groups, yielding a species with a high molar extinction coefficient. After thawing, FBS or a human plasma sample was diluted 4-fold with DTNB to a final concentration of 2 mM in 100 mM ammonium bicarbonate (pH 8.4), followed by a 15 min incubation at room temperature. Absorbance was measured at 412 nm by using a DS-11 FX+ spectrophotometer/fluorometer (DeNovix). Background absorbance from DTNB alone was subtracted. The free thiol concentrations were determined using an l-cysteine calibration curve (ranging from 12.5 to 200 μM) in 100 mM ammonium bicarbonate (pH 8.4). Free thiol concentrations were adjusted to total protein concentrations of FBS and plasma (μmol thiol/g of protein), as measured by the Bradford assay using bovine serum albumin for the standard curve.

### IAA Titration in Serum

To determine the molar excess of IAA needed to block all reactive thiol sites in FBS, a DTNB assay was performed, as described above for free thiol quantitation. In this experiment, however, varying concentrations of IAA from 0.05 to 100 mM (in 100 mM ammonium bicarbonate, pH 8.4) were added to the FBS prior to DTNB addition. After 1 h of incubation at room temperature in the dark, 10 μL of 20 mM DTNB was added to the samples for a final volume of 100 μL. After a 15 min incubation, the absorbance was measured.

### SFN/SFN-*d*_8_ Calibration Curve Generation

SFN calibration standards were prepared from stock (5 nM–1 μM), serially diluted in 225 nM SFN-*d*_8_ in 0.1% formic acid in acetonitrile, and analyzed with the LC–MS/MS MRM Method described above. SFN peak area/SFN-*d*_8_ peak area was regressed against the SFN concentration/SFN-*d*_8_ concentration.

### Testing IAA Rescue of SFN from Thiol Conjugation in Serum

The degree to which IAA can rescue SFN from thiol conjugation in serum was assessed by incubating SFN at 56 and 560 nM for 2 h at room temperature in FBS. After 2 h, 50 μL aliquots were (1) incubated with 50 μL of 50 mM ammonium bicarbonate (pH 8.0) for 45 min at room temperature, (2) incubated with 50 μL of 200 mM IAA (final 1000× molar excess IAA to 0.1 mM FBS thiols) in 50 mM ammonium bicarbonate (pH 8.0) for 45 min in the dark at room temperature, or (3) precipitated immediately with no incubation. Proteins were precipitated (after incubations as described) with 150 μL of ice-cold 0.1% formic acid in acetonitrile with 560 nM SFN-*d*_8_. Samples (*n* = 3) were centrifuged for 5 min at 5200*g*, the supernatant was transferred to an autosampler vial, and SFN was quantified using the SFN/SFN-*d*_8_ area ratio. Standard deviation was determined based on single injections of the three replicate samples. To assess changes in SFN plasma concentration over time, a *t* test was used to compare the 2 h time point for each participant with their 1 h time point and to compare the 3 h time point with the 2 h time point. The method limit of quantitation (LOQ) of SFN in plasma was defined at a signal-to-noise ratio of 10.

### Human Plasma Sample Preparation and Quantitation

A pharmacokinetic study was conducted at Northern Arizona University, approved by the university’s Institutional Review Board (#1641690-1). Subjects were 12 healthy, young (19–30 y), men (*n* = 4), and women (*n* = 8). The mean age was 22 ± 3 years, the mean height was 168 ± 3 cm, the mean weight was 64.9 ± 3.0 kg, the mean body mass index was 23 ± 3 kg/m^2^, and the mean waist circumference was 75 ± 2 cm.

Subjects were asked to refrain from consuming foods high in SFN for 3 days prior to the trial, and a list of foods to avoid was provided. Subjects were also asked not to exercise or perform overly strenuous activity for 24 h prior to the trial. Subjects arrived at the laboratory after an overnight fast, and a baseline blood draw was taken. The subjects then consumed three EnduraCell (Cell-Logic Pty Ltd., Queensland, AUS) capsules with water. As per the manufacturer, each capsule contained 700 mg of 100% whole broccoli sprout powder, including active myrosinase and 21 mg of glucoraphanin, which upon full conversion to SFN would yield ∼8 mg, equaling ∼24 mg of SFN total per three-capsule dose. We note that full conversion to SFN, even with active myrosinase in the supplement, is not expected.^20^ Additional blood draws were taken at 1, 2, and 3 h postconsumption. Whole blood was collected into 6 mL ethylenediaminetetraacetic acid vacutainers and refrigerated for 20 min prior to centrifugation at 1200*g* for 20 min at 4 °C. Plasma was aliquoted and stored at −80 °C, until shipping on dry ice to Villanova University.

For SFN quantitation via LC–MS/MS, plasma was thawed at room temperature for 30 min, vortex mixed briefly, and centrifuged at 12,000*g*, 4 °C for 5 min. Aliquots of plasma (50 μL) from all four-time points were transferred to separate microcentrifuge tubes and incubated with 50 μL of 600 mM IAA (1000× molar excess IAA to 0.6 mM plasma thiols) in 50 mM ammonium bicarbonate (pH 8.0) at room temperature for 45 min in the dark. The plasma samples taken 1 h postconsumption were also processed without IAA addition, precipitating proteins immediately upon thawing. Proteins were precipitated in all samples by the addition of 150 μL of ice-cold 0.1% formic acid in acetonitrile spiked with 40 ng/mL SFN-*d*_8_. Samples were centrifuged for 5 min at 4 °C at 12,000*g*. The supernatant was transferred to autosampler vials and analyzed immediately. All samples were analyzed in triplicate and quantified as described in the LC–MS/MS MRM Method section. Standard deviation was determined based on three replicate injections of a given sample.

## Results

### SFN Metabolite Preparation and Dissociation Testing

We initiated our study by attempting to create an external calibration curve for the quantitation of SFN metabolites SFN-GSH, SFN-NAC, and SFN-Cys in human plasma as previously described.^15^ MS MRM transitions for the metabolites could be optimized by the direct infusion of standards into the mass spectrometer. However, HPLC analysis of individual samples of the metabolites dissolved in 0.1% formic acid in water (pH 3) produced a large free SFN peak, indicating that the metabolites were dissociated (Figure S1). It is unlikely that this large amount of free SFN is an impurity from metabolite synthesis. In addition, a free SFN MRM peak was observed at the metabolite retention time, suggesting that some amount of remaining intact metabolite underwent postcolumn, in-source reduction to free SFN.

We determined the extent to which SFN-GSH (as a representative SFN metabolite) dissociates under HPLC conditions. SFN-GSH standards ranging from 10 mM to 100 nM were prepared in 0.1% formic acid in water (with 10 mM approaching MS detector saturation, resulting in a loss of peak resolution), and free SFN was quantitated. As shown in Table 1, at 10 mM SFN-GSH, 95% of the SFN was present as the unconjugated free form. At 1 μM, SFN had completely dissociated from the SFN-GSH conjugate. Therefore, over the time required to generate an experimental calibration curve, SFN-thiol metabolites can fully dissociate in 0.1% formic acid (pH 3) and are largely dissociated even at 10 mM SFN-GSH, complicating LC–MS/MS method development.

**Table 1:** Percent of Free SFN Present in SFN-GSH Solutions, at SFN-GSH Concentrations Typically Used for Generation of an External Calibration Curve

[SFN-GSH]^a^ (mM)	% free SFN	% SFN-GSH
10	95%	5%
1	96%	3%
0.1	97%	2%
0.01	99%	1%
0.001	100%	0%
0.0001	100%	0%

a The calculated concentration of the SFN-GSH standard prepared in 0.1% formic acid in water (pH 3) on ice. Standards were analyzed for metabolite dissociation immediately after preparation.

### Assessing the Degree of SFN Conjugation to Thiols in Serum

A second issue in the LC–MS/MS quantitation of SFN in human plasma is the tendency of SFN to react with thiols present in plasma proteins. SFN covalently associated with thiols would be lost when samples are precipitated with an organic solvent, thus reducing the percent recovery of SFN. A determined percent recovery of spiked SFN in a matrix such as serum or plasma may thus be quite high if the sample is analyzed immediately after spiking and may decrease when the spiked SFN-matrix is incubated prior to processing for analysis.

**Figure 2:**
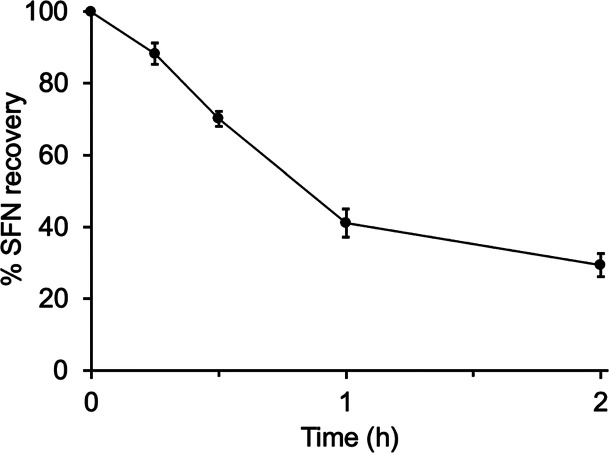
Percent recovery of spiked SFN in serum over time while incubating at room temperature. *N* = 3 replicate injections.

As a proof of the principle of whether SFN is lost over time due to protein thiol binding, a known amount of SFN was added to FBS. Aliquots were analyzed at time points over the course of a 2 h incubation. The SFN peak area of the spiked serum samples was compared with that of an SFN standard of equivalent concentration in 0.1% formic acid in acetonitrile to calculate the percent recovery. SFN percent recovery in FBS decreased substantially with incubation time (Figure 2). After 15 min of incubation, approximately 90% of the SFN was recovered. After 2 h of incubation, SFN loss to thiol conjugation was significant, with approximately 30% recovered.

### Serum and Plasma Free Thiol Measurements and IAA Titration

Given the propensity of SFN-thiol conjugates to dissociate upon dilution in aqueous solutions and the loss of SFN due to conjugation with plasma protein thiols, a method was developed to free SFN from both thiol metabolite conjugates and protein thiols in serum or plasma prior to protein precipitation and analysis. IAA is an electrophile with an iodide leaving group and reacts irreversibly with thiols, forcing SFN conjugates to dissociate by Le Chatelier’s Principle and preventing their reformation (Figure 1B). As a first step, the concentration of free thiols in FBS and a representative human plasma sample was measured by the DTNB assay. DTNB-cysteine and Bradford assay calibration curves are shown in Figures S2 and S3. The concentration of free thiols was 99 ± 7 μM in FBS and 590 ± 10 μM in plasma, in line with reported values of 0.4–0.6 mM total reduced thiols in plasma.^21^ The free thiol/total protein concentration ratios in FBS and plasma were 2.1 ± 0.3 and 7.0 ± 0.7 μmol/g, respectively.

To determine the molar ratio of IAA/thiols required to conjugate all reactive thiol sites in FBS (and later adjusted to plasma thiol concentrations), IAA was titrated into FBS, using DTNB to quantify the free thiols. When all thiols in FBS are occupied, the absorbance at 412 nm is quenched, indicating no free thiols are available to react with DTNB. As shown in Figure S4, a 100 molar excess of IAA to FBS thiols (based upon 0.1 mM thiol concentration, determined above) was sufficient for all FBS thiols to irreversibly react with IAA within 1 h. Translating this to the amount of IAA required for human plasma samples of the same volume, based on ∼6× higher thiol concentration in plasma, 600 molar excess IAA to thiols would be required. A 1000 times molar ratio of IAA/thiols was used going forward prior to protein precipitation to ensure a high percent recovery of SFN.

### SFN/SFN-*d*_8_ Calibration Curve Generation

For all SFN quantitation experiments in this work, a SFN/SFN-*d*_8_ area ratio calibration curve was generated by preparing SFN standards (5 nM to 1 μM) in 225 nM SFN-*d*_8_ in 0.1% formic acid in acetonitrile. Good calibration curve accuracy (<5% bias) for SFN quantitation was achieved with the SFN-*d*_8_ internal standard. Linear fit with 1/*x* weighting factor was used to quantitate SFN. A good fit to this model yielded a high correlation coefficient, *R*^2^ = 1 (Figure S5).

### Testing SFN Rescue with IAA

Due to the high degree of SFN loss observed in serum samples, we next tested the ability of IAA to free SFN from thiols and improve percent recovery. Based on the results in Figure 2, SFN (56 and 560 nM) was incubated in FBS for 2 h at room temperature, a sufficient time for SFN to react with protein thiols. The amount of SFN in the samples was measured by LC–MS/MS using three sample preparation procedures. First, samples were precipitated immediately with SFN-*d*_8_ in 0.1% formic acid in acetonitrile. To test the effect of including IAA, samples were instead diluted 2-fold with 50 mM ammonium bicarbonate (pH 8.0) to 100 mM IAA final concentration (1000-fold excess over the serum thiol concentration) and incubated for 45 min, followed by the addition of SFN-*d*_8_ and precipitation. Finally, to test the effect of dilution only, samples were treated for IAA addition, but IAA was omitted. Free SFN in each sample was determined by comparing it with an SFN standard of equivalent concentration in 0.1% formic acid, using an SFN/SFN-*d*_8_ calibration curve.

First, regardless of the sample preparation method, 99% of the internal SFN-*d*_8_ standard was recovered. This is expected, since samples are precipitated immediately after SFN-*d*_8_ addition, and SFN-*d*_8_ thus had insufficient time to react with thiols, compared to unlabeled SFN that had 2 h to react with serum thiols (see also Figure 2).

**Figure 3:**
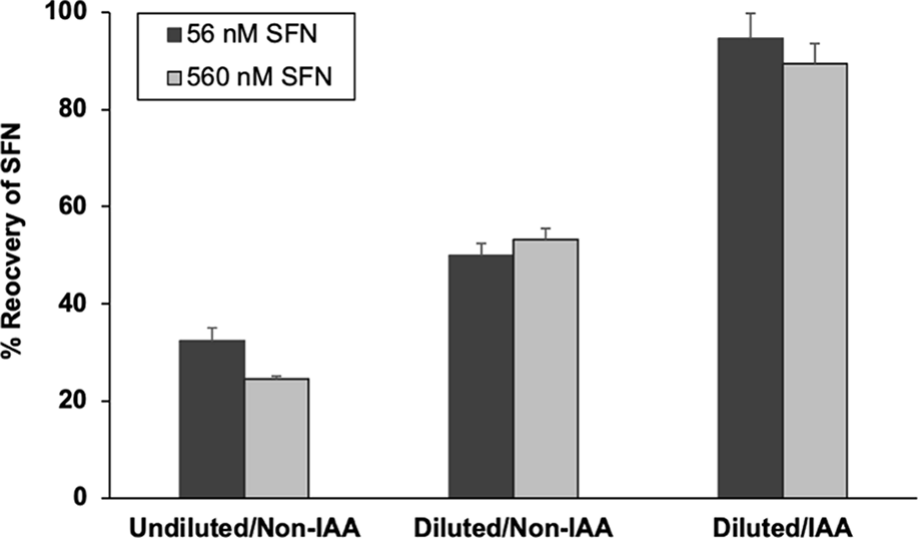
Percent recovery of SFN in serum via LC–MS/MS and the effect of IAA. SFN was incubated for 2 h in FBS prior to workup and analysis. In the “undiluted/non-IAA” method workup, SFN-*d*_8_ internal standard was added, and samples were immediately precipitated and analyzed by LC–MS/MS. Alternatively, the workup differed in that samples were diluted 2-fold in 50 mM ammonium bicarbonate (pH 8.0) with or without IAA and incubated for 45 min prior to addition of internal standard, precipitation, and analysis. *N* = 3 replicate samples.

As shown in Figure 3, recovery of SFN from samples precipitated without the addition of buffer of IAA was 32 ± 3% recovery for the 56 nM sample and 25 ± 1% for the 560 nM in the FBS sample, as expected based on Figure 2. Addition of buffer alone with a 45 min incubation period improved recovery to ∼50%. This was also expected, since dilution alone promotes SFN-thiol dissociation.^6^ The inclusion of IAA further increased recoveries to 94 ± 5% for the FBS sample with 56 nM SFN and to 89 ± 4% with 560 nM SFN. The optimized incubation time with IAA, to allow for full SFN-thiol dissociation while maintaining SFN stability, was determined to be 45 min (data not shown). We note that no SFN-thiol conjugates were detectable in these samples, including any small molecule conjugates. This is expected, for if any were present, they would be precipitated due to the insolubility of SFN metabolites in acetonitrile.^22^ Incubation of the sample with IAA prior to protein precipitation increased the SFN peak area by 6.2-fold (data not shown), permitting a method LOQ for SFN of 4.7 nM, compared to 29 nM in the absence of IAA.

### Plasma Sample Preparation and SFN Analysis

**Figure 4:**
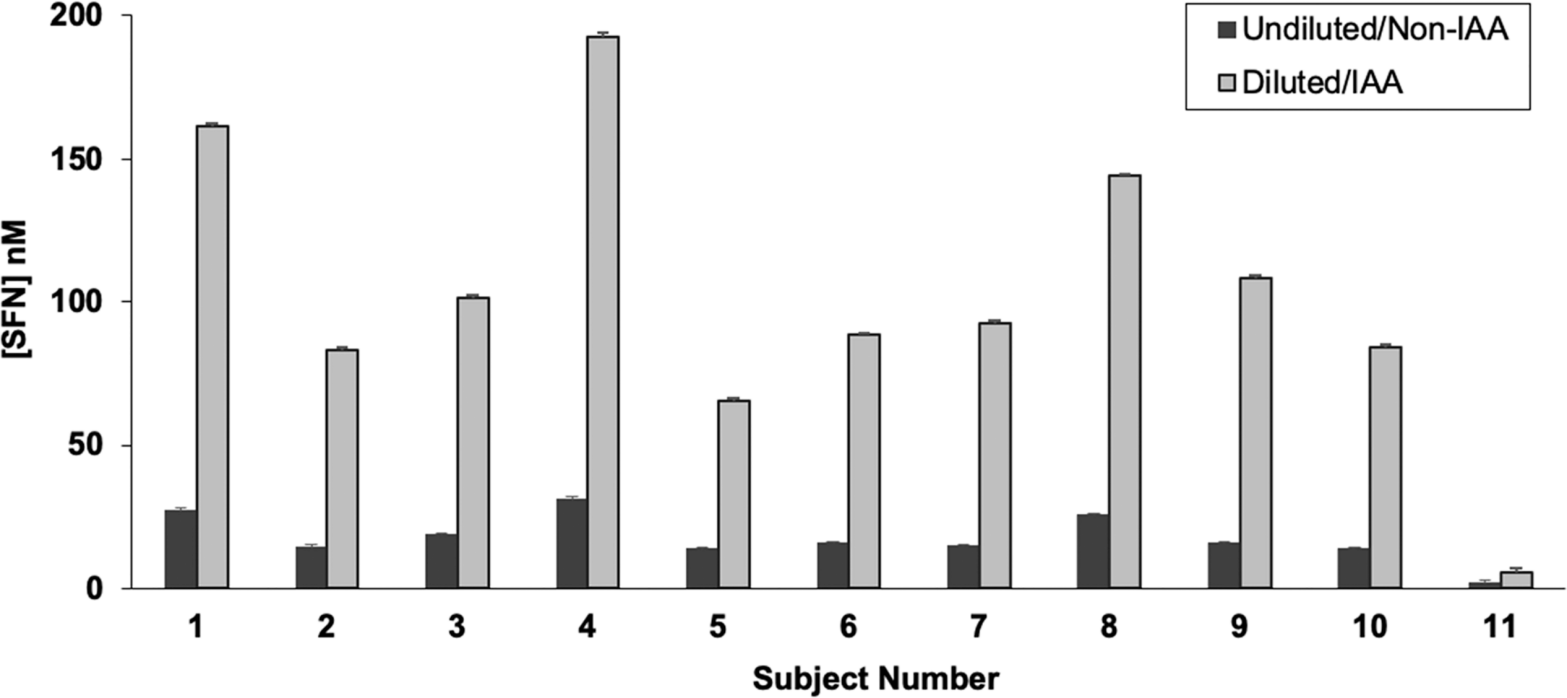
SFN concentrations were measured via LC–MS/MS in plasma samples collected 1 h postconsumption of broccoli sprout powder capsules. Sample were prepared either by dilution with IAA or with no dilution. *N* = 3 replicate injections.

The applicability of the IAA method to clinical plasma samples was assessed. Human plasma samples from 11 participants, 1 h after consumption of broccoli sprout powder capsules, were obtained from an acute SFN supplementation clinical trial. Samples were prepared either by direct protein precipitation with SFN-*d*_8_ in 0.1% formic acid in acetonitrile or by 2-fold dilution with IAA (1000× the plasma thiol concentration) in 50 mM ammonium bicarbonate (pH 8.0) for 45 min at room temperature before protein precipitation. SFN concentrations measured with the IAA method were 6 times higher on average than those measured using the sample preparation that omits IAA incubation (Figure 4).

**Figure 5:**
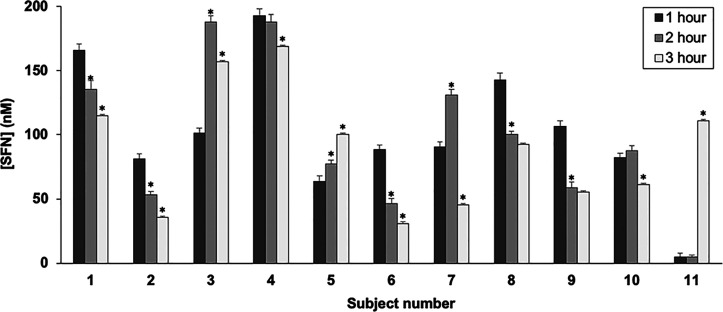
SFN concentrations measured in 11 human plasma samples from an acute broccoli sprout powder supplementation clinical trial. The times in the legend (1, 2, or 3 h) indicate when blood plasma was taken after consumption of the supplement. The asterisks for the 2 and 3 h time points indicate a *p*-value of <0.05, comparing that time point to the previous time point for that participant. No comparisons were done between the 1 h time points and the preconsumption levels, since only two participants had detectable SFN, as described in the main text. *N* = 3 replicate injections.

The IAA incubation method was then used to assess SFN concentrations in human plasma from the same 11 participants preconsumption and 1-, 2-, and 3 h postconsumption. SFN was largely undetected in preconsumption samples, except for participants 4 and 8, where less than 1 nM was detected. Postconsumption, SFN concentrations in plasma increased for all subjects, with the highest being 193 nM, for subject 4, 1 h postconsumption (Figure 5). The profile of SFN detection in plasma over time varied across the subjects. In 6 out of 11 subjects, SFN concentrations peaked 1 h postconsumption and decreased thereafter. For 7 of the subjects, the 3 h levels decreased from earlier time points, indicating clearance of SFN, which is consistent with previous observations.^15,23^ One subject, 11, was an outlier, with an abrupt increase at the 3 h time point. Across all participants, the median plasma level for the 1 h time point was 91 nM. The interquartile range, 61 nM, reflects the varied profile. The median and interquartile range of the 2 h samples were 86 and 80 nM, respectively, and for the 3 h samples were 92 and 67 nM.

## Discussion

A primary finding of this work is that addition of SFN (labeled or unlabeled) to serum/plasma samples or matrices as an internal control during LC–MS/MS sample preparation, followed immediately by protein precipitation, does not adequately capture the loss of SFN due to conjugation to plasma thiols. In addition, the SFN-glutathione conjugate prepared at 10 mM in 0.1% formic acid in water dissociated by approximately 95% to free SFN, illustrating the challenge in preparing thiol metabolite standards. Inclusion of IAA in the sample preparation method frees SFN from both protein thiols and thiol metabolites, resulting in the measurement of total SFN.

Regarding loss of SFN in serum and plasma due to conjugation to protein thiols (which are precipitated and discarded during sample preparation), we observed that approximately 70% of SFN spiked into FBS was lost after 2 h of incubation. The percentage of SFN lost in this manner in human plasma upon protein precipitation would likely be greater than the loss we observed in FBS, as the free thiols in human plasma are approximately 6 times higher than in FBS. This agrees with the reported 81–86% decrease in SFN peak area in SFN spiked blank plasma compared to a SFN standard.^15^ In contrast, approximately 90% of SFN was recovered after only 15 min of incubation, which is in agreement with various previously reported high percent recoveries. Incubation times of SFN in blank serum or plasma are often not reported in the literature. In samples from a clinical trial, SFN would react with protein thiols in plasma in the period from when SFN enters the bloodstream after ingestion until the processing of plasma samples, on a likely time scale of several hours. Thus, a substantial amount of SFN may be unaccounted for during the protein precipitation stage of sample processing prior to LC–MS/MS analysis. The use of IAA to rescue SFN from thiols significantly improves its percent recovery. The LOQ of this method (4.7 nM) is at the low end of the range of those previously reported for SFN in human plasma, e.g., 7.8–20.8 nM.^12,15,18,24^

Acidic conditions have been reported to significantly stabilize SFN metabolites.^6,12^ However, we find that at pH 3 (0.1% formic acid) 10 mM SFN-GSH largely dissociated within minutes. In a personal communication from Toronto Research Chemicals (the supplier of SFN and metabolite standards), SFN metabolites were described as “unstable in HPLC conditions”, preventing purity assessment via HPLC. A benefit of the IAA method is that it circumvents the difficulties in the LC–MS/MS method development associated with the facile dissociation of SFN-thiol metabolites.

The IAA method complements the panel of methods available to quantitate SFN and its metabolites in clinical samples. Because the IAA method quantitates total SFN, information about the metabolic profile is lost, which could be important if the bioactivities of SFN-thiol metabolites differ from each other. The gold standard for metabolite detection is isotope-dilution mass spectrometry,^18,22^ in which d8-SFN and thiol conjugates are diluted into analyzed samples to correct for losses, including loss of the SFN conjugates during dilution and other stages of sample preparation. Somewhat analogous to the IAA method, in the past, the cyclo-condensation assay was used extensively to quantitate total isothiocyanates and thiol conjugates by cyclo-condensing with 1,2-benzenedithiol to produce 1,3-benzodithiole-2-thione, detected by UV spectroscopy upon HPLC separation.^25,26^ One potential complication of this method is that the other chemicals possibly present in plasma can also generate the detected product.^26^ The method also requires the protein precipitation of plasma samples prior to analysis. Of general note in clinical sample analysis is that detecting SFN and its metabolites in urine samples does not require a protein precipitation step, given the usually low protein content in urine. Thus, loss of SFN to precipitation of protein-thiol conjugates is a nonissue in urine samples. (SFN and its metabolites are also at much higher concentrations in urine, further simplifying analysis.) Urine analysis combined with plasma analysis reveals a complete pharmacokinetic profile of bioavailable SFN, as SFN and its metabolites are rapidly excreted in urine.^25^ Together, the various methods provide a comprehensive approach for assessing total SFN, total isothiocyanates, and the metabolic profile of SFN-thiol conjugates in biological fluids used for clinical analysis.

In summary, because of the facile reversibility of SFN-thiol conjugates under physiological conditions, both conjugates and free SFN are bioactive. Because the IAA method detects total SFN, whether it is free, conjugated to protein thiols, or a mercapturic acid pathway metabolite, it detects bioavailable SFN in plasma. The IAA method also requires minimal sample preparation and manipulation prior to injection compared with other SFN detection methods. Overall, IAA simplifies the measurement of bioavailable SFN in plasma, streamlining the analysis for in vivo and clinical trials.

## Supporting Information Available

MRM transitions; dissociation of SFN metabolites in 0.1% formic acid; l-cysteine calibration curve; Bradford calibration curve; IAA titration in FBS; and the SFN/SFN-*d*_8_ area ratio calibration curve (PDF)

## Abbreviations

(CE): collision energy
(DP): declustering potential
(DTNB): 5,5′-dithio-bis (2-nitrobenzoic acid)
(ESI): electrospray ionization
(FBS): fetal bovine serum
(GSH): glutathione
(IAA): iodoacetamide
(LOQ): limit of quantitation
(LC–MS/MS): liquid chromatography–tandem mass spectrometry
(MRM): multiple reaction monitoring
(NAC): N-acetyl cysteine
(SFN): sulforaphane
(SPE): solid-phase extraction
(TFA): trifluoroacetic acid
