# Supplementary material for: Bioavailable Sulforaphane Quantitation in Plasma by LC–MS/MS Is Enhanced by Blocking Thiols
Source: J Agric Food Chem. 2023 Aug 16;71(34):12875–82. doi: 10.1021/acs.jafc.3c01367 (PMC10472501; doi:10.1021/acs.jafc.3c01367)
Supplement: Supplementary file 1 — jf3c01367_si_001.pdf [file jf3c01367_si_001.pdf]

Supporting Information

Bioavailable sulforaphane quantitation in plasma by LC-MS/MS is enhanced by blocking thiols

Rachel S. Grady[a], Tinna Traustadóttir[b], Anthony F. Lagalante[a], Aimee L. Eggler[a]*

[a] Department of Chemistry, Villanova University, Villanova, Pennsylvania, 19085, USA
[b] Department of Biological Sciences, Northern Arizona University, Flagstaff, Arizona, USA

*Corresponding author.

Table S1. Optimized MRM transitions for the quantitation of SFN and its metabolites and analyte retention times.

Compound	MRM Transition	DP (V)	CE (V)	Retention Time (min)
**SFN**	178>114	30	16	6.77
**SFN-d_8**	185.8>122.2	30	16	6.774
**SFN-GSH**	485.3>162	31	85	5.64
**SFN-NAC**	341.1>178	18	55	5.95
**SFN-Cys**	299>136	15	40	5.92

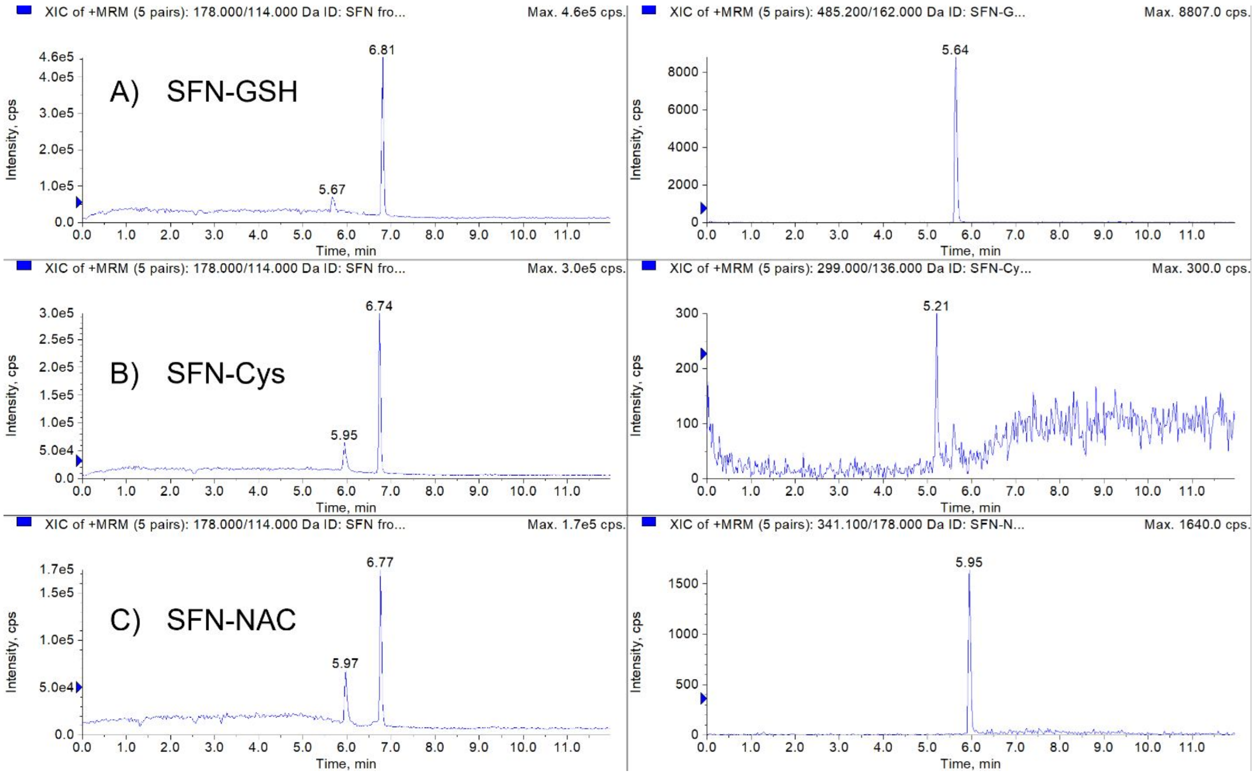

Figure S1. The dissociation of SFN metabolites in 0.1% formic acid in water. A) SFN-GSH, B) SFN-Cys, and C) SFN-NAC. The extracted ion chromatogram (XIC) for free SFN is shown in the left pane, and the conjugated metabolite XIC is shown in the right pane for each compound.

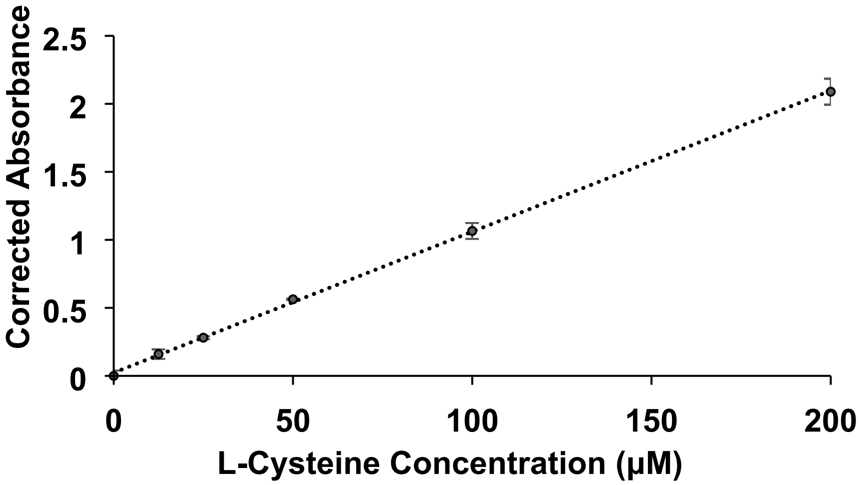

Figure S2. L-cysteine calibration curve. Absorbance was measured at 412 nm to quantitate DTNB-cysteine conjugation (n=3 measurements of a given concentration).

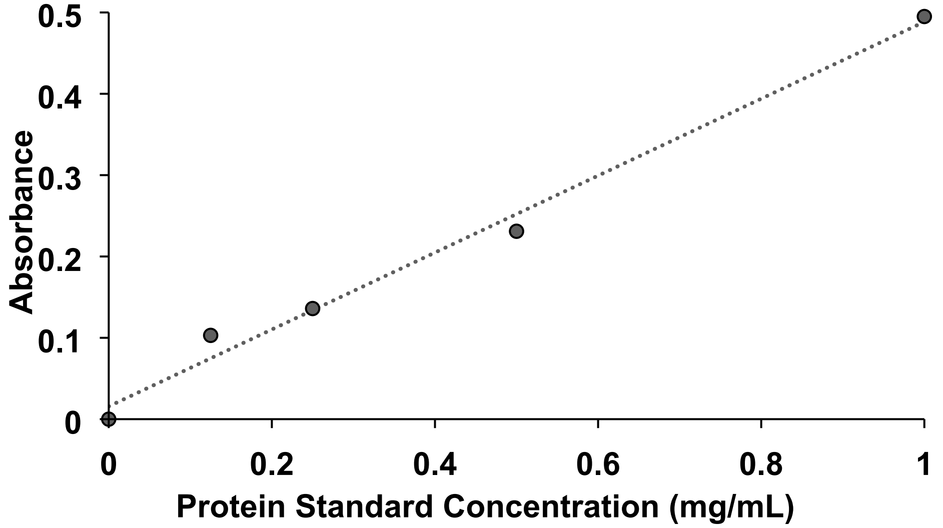

Figure S3. Bradford calibration curve. Absorbance was measured at 595 nm after addition of Bradford reagent to the bovine serum albumin protein standard (n=1).

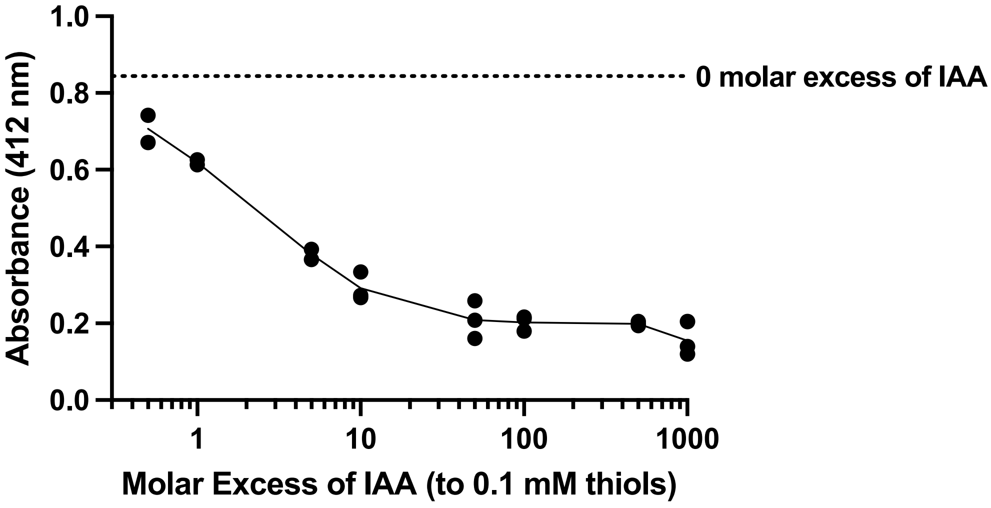

Figure S4. IAA titration curve generated by incubating FBS with 0.05-100 mM IAA (0.5 to 1000 times molar excess of IAA to free thiols in FBS). A DTNB assay was used to determine the molar excess amount of IAA that binds to all free thiols in FBS, by observing a quenching in absorbance at 412 nm. Replicates shown are multiple measurements from the same sample.

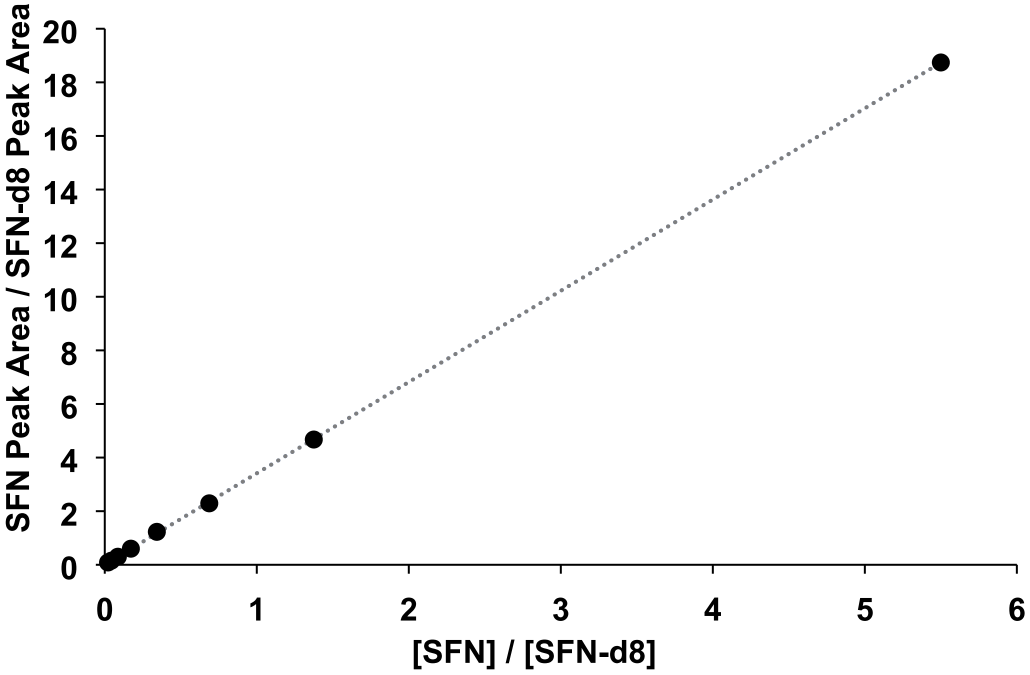

Figure S5. SFN/SFN-d_8 area ratio calibration curve (n=1).
